# Glucose alters the evolutionary response to gentamicin in uropathogenic Escherichia coli

**DOI:** 10.1099/mic.0.001548

**Published:** 2025-03-28

**Authors:** Shalini Choudhary, Jacob A. Smith, Alan McNally, Rebecca J. Hall

**Affiliations:** 1 Department of Microbes, Infection and Microbiomes, School of Infection, Inflammation and Immunology, College of Medicine and Health, University of Birmingham, Birmingham, B15 2TT, UK

**Keywords:** antimicrobial resistance, gentamicin, glucose, uropathogenic *Escherichia coli*

## Abstract

Urinary tract infections (UTI) are a major health and economic concern. Uropathogenic *Escherichia coli* (UPEC) are the leading cause of UTI, and antibiotic-resistant UPEC are increasingly common. The microenvironment of the urinary tract is metabolically distinct, and there is growing interest in understanding the extent to which metabolism may influence UPEC infection and response to antibiotics, and how this varies between individuals. Diabetes, characterized in part by glycosuria, is a known risk factor for UTI and is associated with more severe infections. The role that glucose plays in driving UPEC evolution remains unclear. Through experimental evolution with a single UPEC isolate, we identified mutations in the RNA polymerase sigma factor *rpoS* associated with long-term glucose exposure. We found that the presence of the antibiotic gentamicin resulted in mutations in genes including *trkH*, which encodes a potassium ion uptake system previously linked to aminoglycoside resistance, and in the autotransporter *hyxB*. Strikingly, these mutations were not present in populations exposed to a combination of both glucose and gentamicin. This suggests that glucose may influence the survival of mutants in gentamicin, providing new avenues for understanding the evolution and treatment of UPEC-mediated UTI in high-risk individuals.

## Data availability

The DNA dataset generated and analysed during the current study is available from NCBI with BioProject accession PRJNA1178604. Individual accession numbers are provided in Table S1, available in the online version of this article.

## Introduction

Urinary tract infections (UTI) are a major cause of morbidity and mortality globally [1,2]. Certain demographics are disproportionately affected, including women and individuals with indwelling catheters or diabetes [3,6], and recurrent infections are common [3]. UTI are treated by a panel of antibiotics depending on the acuteness and severity of infection, and whether it has progressed to the bloodstream causing urosepsis [7,8]. Uropathogenic *Escherichia coli* (UPEC) are the leading cause of UTI, with antibiotic and multidrug-resistant UPEC strains increasingly common [7,9,11].

While virulence factors are known to contribute to UPEC pathogenicity [2,12,14], less is known about the extent to which other genes and pathways influence infection, evolution and antibiotic resistance. The urinary tract is uniquely composed of metabolites including urea, uric acid, ammonia and creatinine, thereby creating a distinct metabolic microenvironment to which UPEC are exposed [15,16]. The concentration of glucose in urine can also become elevated (glycosuria, defined as greater than 0.25 mg ml^−1^ [17]) as a result of conditions including diabetes mellitus and gestational diabetes. Genes involved in glucose transport and metabolism have been shown to be important for UPEC colonization of the urinary tract, including *ptsG* (a component of a glucose-specific phosphotransferase system) and *pgi* (catalyses the reversible reaction between glucose 6-phosphate and fructose 6-phosphate) [16,18,20]. Glucose has also been found to alter virulence gene expression in UPEC strains [21]. Given the increased risk of UTI amongst this demographic [22], it is important to understand the extent to which glucose might influence antibiotic susceptibility or drive the evolution of resistance in UPEC.

Using a clinical UPEC strain, we identified that the presence of glucose reduced the impact on the growth of a sub-inhibitory (25% MIC) concentration of gentamicin, an aminoglycoside used in the UK to treat severe UTI or urosepsis [7,8]. We then experimentally evolved the UPEC strain in the presence of either glucose, gentamicin or both glucose and gentamicin. Analysis of short read sequences of strains from the evolved populations showed that glucose exposure led to mutations in the RNA polymerase sigma factor *rpoS*, gentamicin to mutations in the autotransporter *hyxB* and the potassium ion uptake system *trkH*. Crucially, no mutations in *trkH* nor *hyxB* were identified in the populations that evolved in both glucose and gentamicin. While this study uses only a single UPEC isolate, our data suggest that metabolism may play a role in the treatment and evolution of UPEC-associated UTI.

## Methods

### Strains and growth conditions

*E. coli* USVAST002, originally isolated from a human urine sample [23], was used throughout the study. Long and short reads for USVAST002, plus short reads of all evolved strains, are available from NCBI with BioProject accession PRJNA1178604. Individual accession numbers are provided in Table S1. Strains were grown in Luria Bertani (LB) broth (E and O Laboratories) unless specified.

### Bacterial growth assays

To measure the potential effect of glucose on antibiotic sensitivity, USVAST002 was first streaked from a glycerol stock onto an LB agar plate and incubated overnight at 37 °C. A single colony was used to inoculate 5 ml LB broth in a 30 ml universal before overnight incubation at 37 °C with 180 r.p.m. agitation. The culture was then diluted 1:1,000 in LB. Two times final concentrations of glucose (2 mg ml^−1^) and gentamicin (500, 250 and 100 ng ml^−1^) were added to LB, 50 *µ*l of which was then added to each test well of a 96-well plate in technical triplicate, followed by 50 *µ*l of the dilute cell suspension to a final volume of 100 *µ*l per well. Glucose- and gentamicin-free conditions were also included. Once candidate evolution concentrations were selected, the assay was repeated with five independent colonies per condition in a fresh batch of LB with a randomized plate layout. Plates were incubated for 24 h in a microplate reader (Tecan) at 37 °C with continuous shaking at 162 r.p.m., with absorbance measurements (OD600) taken every 30 min.

### Minimum inhibitory concentration assays

MIC of the ancestral and each of the sequenced evolved strains were estimated in U-bottom 96-well plates [24]. For this, overnight cultures were diluted 1:2,000 in Iso-Sensitest broth (ISB) (Thermo Fisher Scientific) and incubated in triplicate in seven different concentrations of gentamicin ranging from 16 *µ*g ml^−1^ to 250 ng ml^−1^, including a non-antibiotic control. MICs were recorded following an 18 h incubation at 37 °C, with significance considered if results were two doubling dilutions away from the ancestor.

### Experimental evolution

The ancestral *E. coli* USVAST002 isolate was streaked from a glycerol stock onto an LB agar plate and incubated overnight at 37 °C. Microcosms were made in 30 ml universals with 5 ml LB, each inoculated with a single colony from the ancestor plate. There were six independent biological replicates per condition: LB no antibiotic (henceforth ‘control’); LB+2 mg ml^−1^ added glucose (‘Glc’); LB+500 ng ml^−1^ gentamicin (‘GEN’); LB+2 mg ml^−1^ glucose+500 ng ml^−1^ gentamicin (‘Glc+GEN’). Microcosms were incubated for 24 h at 37 °C with 180 r.p.m. agitation, before a 1% transfer of cell suspension into fresh media. This 1% transfer was repeated every 24 h for 21 days. Every seventh day, the whole population was centrifuged at 3,600 r.p.m. (Thermo Scientific Megafuge 40 R TX-1000) for 5 min, resuspended in 1 ml 50% glycerol and stored at −80 °C.

### Genome sequencing

Sequencing of the ancestor (hybrid) and evolved (short read) strains was performed by MicrobesNG (UK). A single colony of the ancestral strain was sequenced from pure culture. For the six control populations, a single colony was randomly selected. To select colonies for sequencing from the Glc, GEN and GLC+GEN populations, the populations were first grown to single colonies on an LB agar plate before 16 colonies per population were added directly to 100 *µ*l of 1 *µ*g ml^−1^ gentamicin in LB in individual wells of a 96-well plate. The plate was then incubated for 24 h at 37 °C with 162 r.p.m. agitation and the resulting kinetics were plotted. From this assay, three strains per population were selected for sequencing to represent the range of growth curve profiles, aiming to capture greater genetic diversity within the population than could be observed from a single colony. The growth assay was then repeated in triplicate for each of the selected strains, and these strains were used in all subsequent assays. Full details of each of the sequenced strains, including accession numbers, are provided in Table S1.

### Bioinformatics

The ancestor hybrid assembly was generated using Flye (v2.8.1-b1676) [25], polished using Polypolish (v0.5.0) [26] and annotated with Bakta (v1.8.2) [27]. Genes of interest that were assigned a function but not a gene name were confirmed by BLASTn queries. Variants were called against the assembled ancestor using breseq (v0.36.0) [28] with a consensus minimum variant coverage of ten and a minimum mapping quality of 20. Genes with variants that were present in the control populations were discounted from the test populations. Resistance genes in the ancestral strain were predicted using ABRicate (v0.8) to query the ResFinder database, and sequence type was ascertained using mlst (v2.23.0) [29].

### Statistical analyses

All analyses were conducted in Python (v3.9.10). Area under the curve measurements were calculated using numpy.trapz (v1.22.3). Significance testing was conducted using pair-wise one-way ANOVA tests with the SciPy (v1.8.0) f_oneway function. The Bonferroni correction was used to adjust for multiple comparisons. All *P*-values are provided in Supplementary Data sheet 1.

## Results

### Glucose reduces the effect of sub-inhibitory gentamicin on growth

**Fig. 1.**
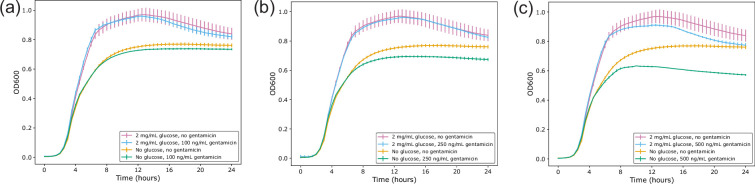
Growth kinetics of USVAST002 in the presence or absence of 2 mg ml^−1^ added glucose plus (**a**) 100 ng ml^−1^, (**b**) 250 ng ml^−1^ or (**c**) 500 ng ml^−1^ gentamicin. Curves for LB only and LB+glucose are the same for each plot and are shown on each for ease of comparison. Measurements in technical triplicate, error bars standard deviation. Corresponding area under the curve plots are provided in Fig. S1. Repeats of (**c**) with five independent biological replicates per condition are provided in Fig. S2.

USVAST002 (ST73) was selected for its status as a UPEC strain with no predicted functional resistance genes. The MIC of gentamicin in ISB against USVAST002 in the absence of added glucose was 2 µg ml^−1^. To assess how the action of gentamicin is affected by the presence of glucose [30], USVAST002 was grown in 2 mg ml^−1^ glucose plus subinhibitory gentamicin [100 ng ml^−1^ (5% MIC), 250 ng ml^−1^ (12.5% MIC) or 500 ng ml^−1^ (25% MIC)]. In the absence of glucose, at increasing concentrations of gentamicin, there was a corresponding reduction in growth (Fig. 1). In the absence of gentamicin, the addition of glucose increased growth significantly (Figs 1, S1 and S2, available in the online Supplementary Material; one-way ANOVA, Bonferroni corrected). The reduction in growth caused by gentamicin in the presence of glucose was smaller than the effect in the absence of glucose. This suggests that glucose acts to reduce the effect of gentamicin.

### Phenotypic changes following prolonged gentamicin exposure

To assess how glucose might affect the evolution of gentamicin resistance, we performed a 21 day evolution experiment in conditions of 2 mg ml^−1^ glucose (Glc), 500 ng ml^−1^ gentamicin (GEN) and both glucose and gentamicin (Glc+GEN). We then screened the growth kinetics of three colonies from each of the evolved populations in LB only and in 1 *µ*g ml^−1^ gentamicin, as well as performing MIC assays in a gradient of gentamicin. In these conditions, the majority of the strains from the Glc and the Glc+GEN populations had comparably worse growth than the ancestor (Figs S3 and S4). In contrast, with the exception of strain GEN 4C, most of the strains from the GEN populations grew comparably to the ancestor in LB only, but six strains grew significantly better in 1 *µ*g ml^−1^ gentamicin (one-way ANOVA, Bonferroni corrected). GEN 4C also grew more poorly than the ancestor in the presence of antibiotics. No strain had an MIC that was significantly different from the ancestor (Table S1).

### Long-term exposure to glucose causes significant genetic changes

**Fig. 2.**
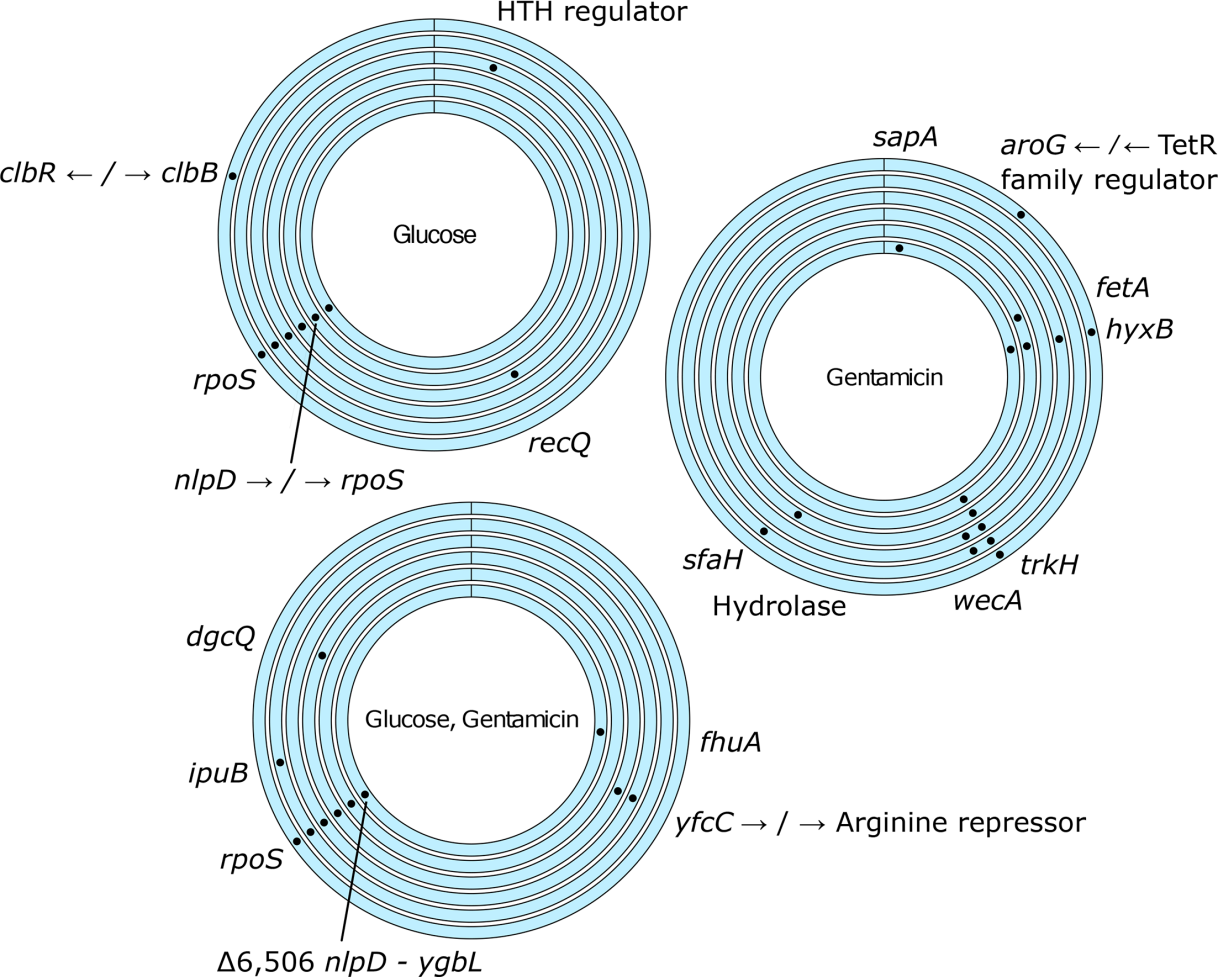
Genetic changes identified in response to Glc, GEN or both Glc+GEN. Each ring represents pooled changes from three individual colonies from each replicate population. Changes also identified in any control population are not presented here.

The extent to which glucose may act as a selective pressure is not understood fully. To investigate this, we performed short-read sequencing of the three individual colonies from each of the evolved populations and analysed the significant genetic changes. The changes were pooled for each replicate to gain a representative understanding of mutations that were present in the population as a whole (Fig. 2) but are provided at an individual strain level in Table S1. Mutations in the RNA polymerase sigma factor *rpoS* were identified in five of the six replicate populations in both the Glc and the Glc+GEN conditions. This included five different non-synonymous SNPs that introduced premature stop codons (Glc P1 Q59*, and Glc +GEN P1 Q251*, P3 Q257*, P4 L30*) (Table S1). In the sixth Glc population, there was an intergenic SNP between *nlpD* and *rpoS*, and in the sixth Glc+GEN, a 6506 bp deletion spanned the same region. No changes were observed in this region in any of the control or GEN populations, indicating that they may have arisen as a result of glucose exposure.

### Glucose alters the evolutionary response to gentamicin

**Fig. 3.**
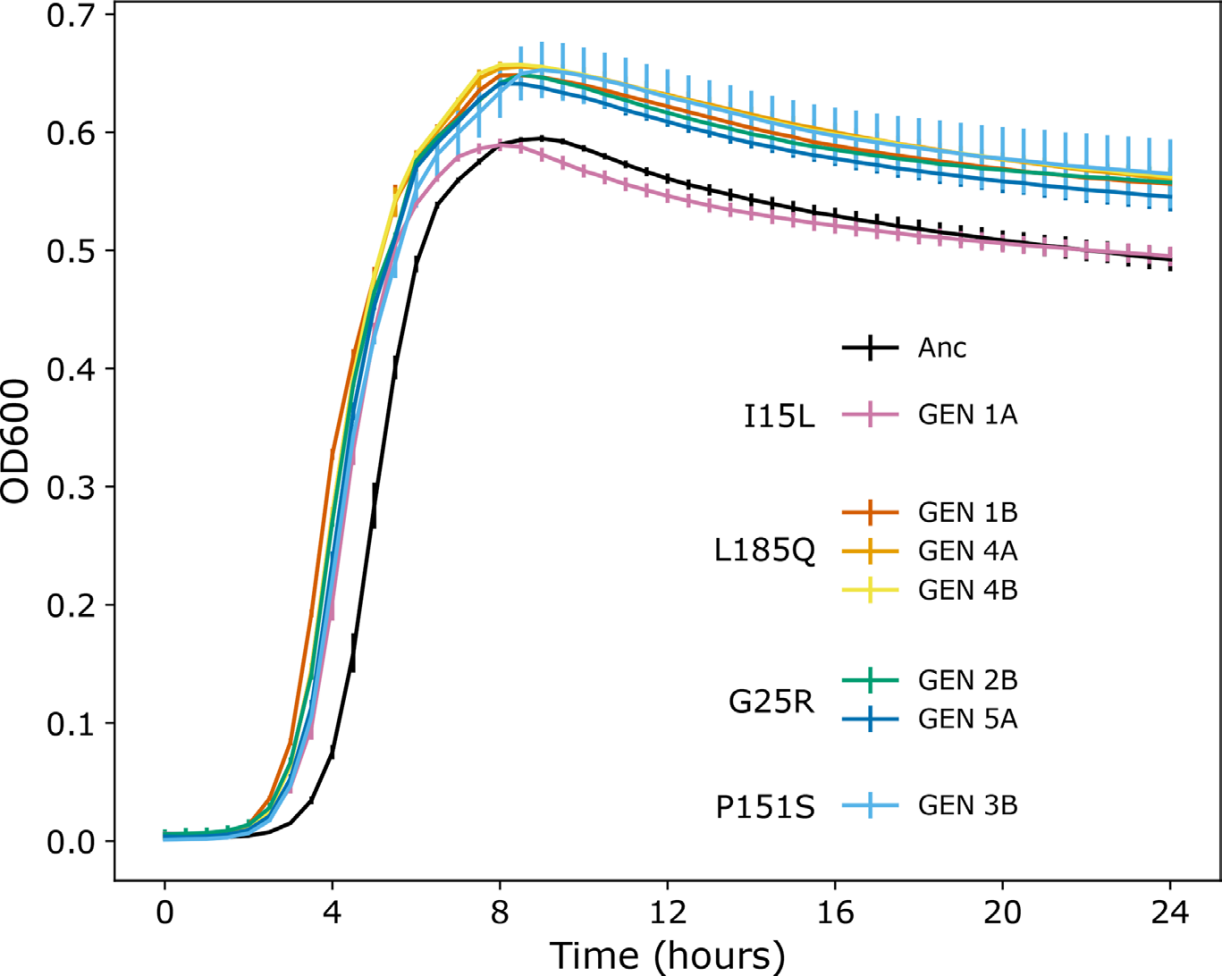
Growth kinetics in 1 *µ*g ml^−1^ gentamicin for the ancestor (Anc) and the strains with mutations in *trkH*. The amino acid change as a result of the mutation in each strain is provided to the left of the key. All curves are presented in Fig. S3, and area under the curve values are presented in Fig. S4. Measurements in technical triplicate, error bars standard deviation.

We then analysed the populations that evolved in the presence of gentamicin to identify any significant genetic changes arising as a result of antibiotic exposure. We identified SNPs in *trkH*, encoding a potassium ion uptake system, in five of the six GEN populations (Fig. 2). There were four different non-synonymous mutations identified across the replicates (I15L, L185Q, G25R and P151S), with one GEN population carrying at least two different mutations (GEN 1A I15L and GEN 1B L185Q) (Table S1). We also found mutations in *hyxB*, an autotransporter, in four of the six populations, and two in *wecA* (involved in enterobacterial common antigen and O-antigen biosynthesis). Strains with three of these *trkH* mutations reached a higher maximum OD than the ancestor in the presence of 1 *µ*g ml^−1^ gentamicin (Fig. 3). In contrast, none of the strains carrying mutations in *hyxB* (GEN 1C, 3A, 3C, 5B, 5C, 6C) had better growth than the ancestor in 1 *µ*g ml^−1^ gentamicin (Fig. S3). Even though mutations in *trkH* and *hyxB* were found within the same GEN population reproducibly, to the resolution of our sequencing (three colonies per population), we did not find any instances of both *trkH* and *hyxB* variants within the same sequenced strain (Table S1). Strikingly, none of the mutations identified in any of the strains from the GEN populations were found in any strains from the Glc+GEN populations. This suggests that the presence of glucose alters the evolutionary response of UPEC to gentamicin.

## Discussion

Given the distinct metabolic microenvironment of the urinary tract and the extent to which it varies as a result of factors including diabetes mellitus and gestational diabetes, very little is known about how the concentration of different metabolites may drive UPEC evolution and response to antibiotics. Here, we discovered that glucose alters the evolutionary response of a UPEC strain to gentamicin.

We identified several genes that were mutated in the presence of gentamicin only, including *trkH* and *hyxB*. The *trkH* gene encodes a potassium ion uptake system [31], and mutations in this gene have been observed in *E. coli* populations adapted to aminoglycosides, resulting in decreased aminoglycoside susceptibility accompanied by increased susceptibility to nalidixic acid and tetracycline [32]. This is in contrast to what we have observed here, whereby strains with mutations in *trkH* did not have significantly different gentamicin susceptibility compared with the ancestor. Indeed, we found that no evolved strain had a significantly different gentamicin MIC compared with the ancestral strain. It is, however, important to note that the evolution medium (LB) differs from the MIC medium (ISB). The initial inoculum size was also smaller in the MIC assays compared with the growth assays, which may impact our conclusions. The *hyxB* gene (also called *upaB* [33]) encodes a virulence-associated autotransporter that mediates adherence to extracellular matrix proteins [33,35]. It has been identified across *E. coli* pathotypes [34], with reports suggesting it is disrupted or absent in diarrhoeagenic *E. coli* but present in nosocomial, but not community, UPEC strains [36,38]. The presence of both *trkH* and *hyxB* mutations in the same populations but, to the resolution of our sampling, never the same genome suggests that there may be selection for only one variant per strain.

Variations within or around the *rpoS* gene were identified in all 12 of the Glc and Glc+GEN populations, and not in either the control or the GEN populations. RpoS is a general stress sigma factor that is known to be induced during glucose starvation [39], with a role in osmotolerance [40]. RpoS also has a role in carbon metabolism, including in the uptake of glucose [41,43]. The lack of *rpoS* mutations in the GEN condition supports a recent study that found that expressing high levels of *rpoS* had minimal impact on antibiotic resistance [44]. From our observations, we could speculate that these mutations in *rpoS* might be in response to a glucose-induced stress, or may work to influence glucose uptake or metabolism. Mutations in *rpoS* have been identified in other evolution experiments, including those investigating the impact of multidrug resistance plasmids on *E. coli* [45] and other clinical enterobacteria [46], as well as on *Pseudomonas aeruginosa* biofilms exposed to imipenem [47]. This may indicate a more generalized stress response.

Our data suggest that glucose may impact the emergence of mutations linked to resistance, possibly due to increased efflux activity reducing the internal antibiotic concentration. AcrD is thought to efflux aminoglycosides in *E. coli* [48,49], but analysis of a highly similar AcrD in *Salmonella* was found to not efflux gentamicin [50]. More research is, therefore, needed to understand the mechanism underpinning these results, including characterizing the effect of *trkH*, *hyxB* and *rpoS* mutations. Our study also used only a single *E. coli* strain which does not account for variations in genetic background, and so further work encompassing the phylogenetic diversity of UPEC should also be considered.

## Supplementary material

10.1099/mic.0.001548 Supplementary Material 1.

10.1099/mic.0.001548 Supplementary Material 2.

## Abbreviations

ISB: Iso-Sensitest broth
LB: Luria Bertani
UPEC: uropathogenic *Escherichia coli*
UTI: urinary tract infections
