# Supplementary material for: Glucose alters the evolutionary response to gentamicin in uropathogenic Escherichia coli
Source: Microbiology (Reading). 2025 Mar 28;171(3):001548. doi: 10.1099/mic.0.001548 (PMC12282228; doi:10.1099/mic.0.001548)
Supplement: Supplementary Material 2. [file mic-171-01548-s002.pdf]

Supplementary

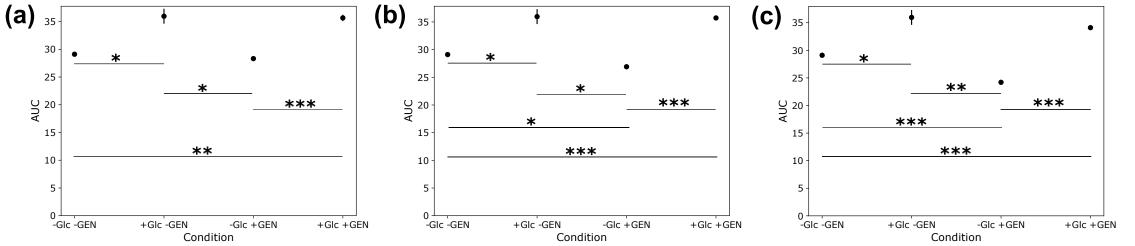

Fig. S1 Area under the curve (AUC) values of growth kinetics in Fig. 1 for USVAST002 grown in the presence or absence of 2 mg/mL glucose plus (a) 100 ng/mL, (b) 250 ng/mL, or (c) 500 ng/mL gentamicin. Measurements in technical triplicate, error bars standard deviation. * $p < 0.05$, ** $p < 0.01$, *** $p < 0.001$, one-way ANOVA, Bonferroni corrected.

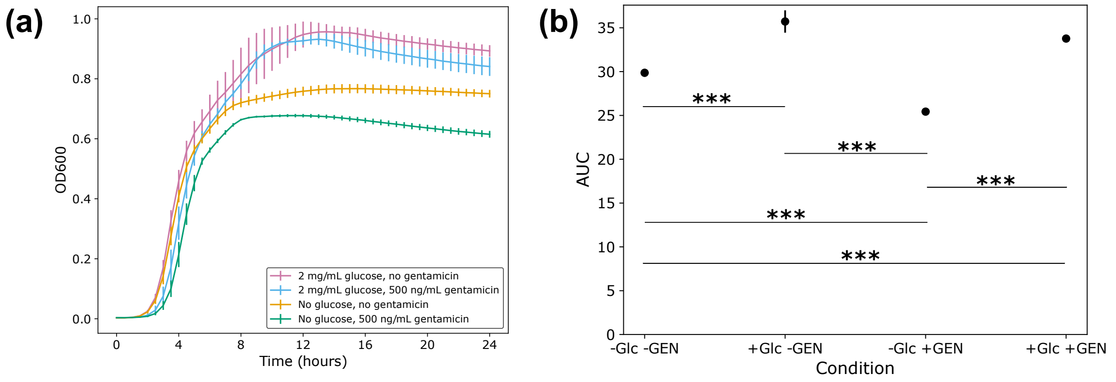

Fig. S2 Growth kinetics (a) and corresponding area under the curve (AUC) values for USVAST002 grown in the presence and absence of 2 mg/mL glucose and the presence and absence of 500 ng/mL gentamicin. Measurements in five independent biological replicates per condition, error bars standard deviation. *** $p < 0.001$, one-way ANOVA, Bonferroni corrected.

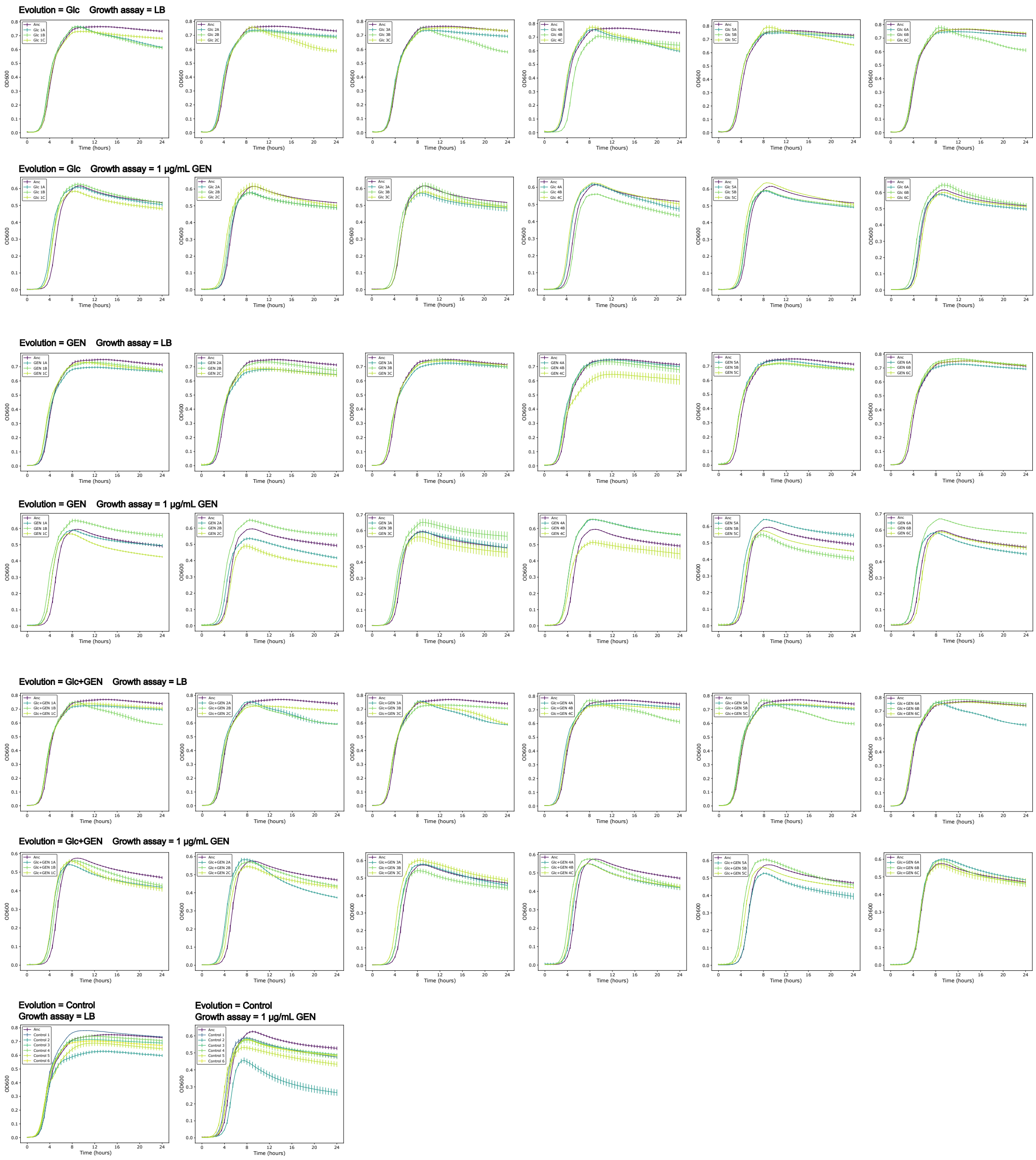

Fig. S3 Growth kinetics of all sequenced strains in LB and in LB + 1 μg/mL gentamicin. Plots are grouped by evolution condition (Glc, GEN, Glc+GEN, Control) and growth assay condition (LB, 1 μg/mL GEN). Measurements in technical triplicate, error bars standard deviation.

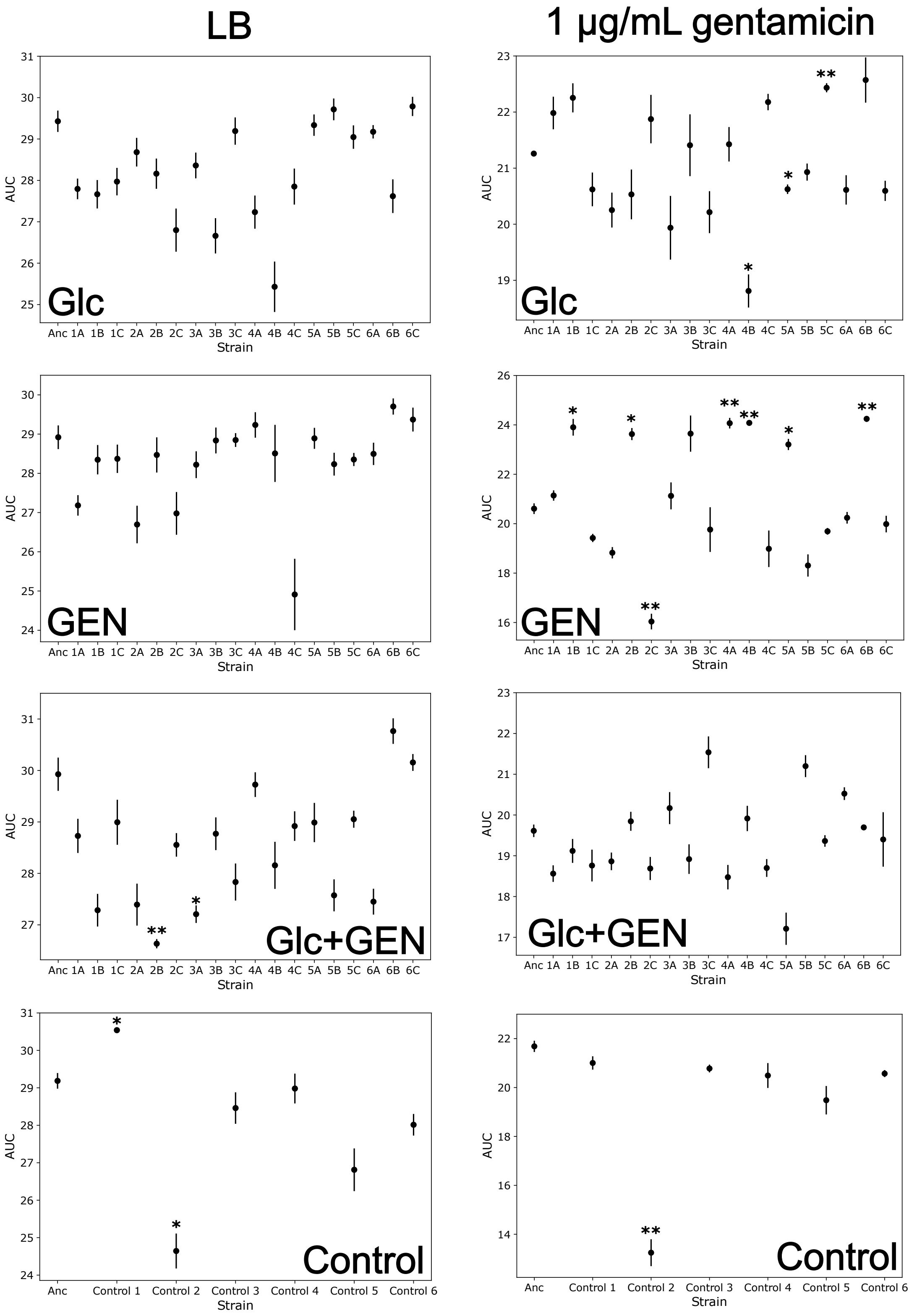

Fig. S4 Area under the curve (AUC) values of growth kinetics in Fig. S3 in LB only (left) or 1 μg/mL gentamicin. Measurements in technical triplicate, error bars standard deviation. * $p < 0.05$, ** $p < 0.01$, one-way ANOVA, Bonferroni corrected.

Table S1. All predicted mutations in the control and test populations, and their gentamicin minimum inhibitory concentrations (MIC).
MIC values mode of triplicate. For all test populations, three independent colonies (A-C) were sequenced. - = no predicted mutation, Glc = glucose, GEN = gentamicin, Chr = chromosome

Condition	Population	MIC (µg/mL)	Contig	Position	Mutation	Annotation	Gene	Accession
Ancestor	-	2	-	-	-	-	-	SRR31135999, SRR31135528
Control	1	4	-	-	-	-	-	SRR31135527
Control	2	2	Chr	1,714,698	T→G	*36Y (TAA→TAC)	*fimA* ←	SRR31135516
Control	2		Chr	3,576,017	A→T	E14V (GAA→GTA)	*ygcU* →	
Control	2		Chr	3,593,195	T→C	L15P (CTA→CCA)	*ftsB* →	
Control	3	2	-	-	-	-	-	SRR31135505
Control	4	2	Chr	66,911	Δ1 bp	coding (873/1542 nt)	*tyrR* ←	SRR31135494
Control	5	4	Chr	785,954	G→A	P150L (CCA→CTA)	*aroG* ←	SRR31135483
Control	6	2	Chr	1,714,699	T→G	*36S (TAA→TCA)	*fimA* ←	SRR31135472
Control	6		Chr	4,375,083	C→T	intergenic (+70/-323)	*Transcriptional regulator* → / → *Transposase*	
Glc	1A	2	Chr	3,598,595	C→T	Q59* (CAG→TAG)	*rpoS* →	SRR31135470
Glc	1B	4	Chr	3,598,595	C→T	Q59* (CAG→TAG)	*rpoS* →	SRR31135469
Glc	1C	2	Chr	4,382,445	(GTATCTGT)$_{6\rightarrow7}$	intergenic (-68/-334)	*clbR* ← / → *clbB*	SRR31135468
Glc	2A	2	-	-	-	-	-	SRR31135526
Glc	2B	2	-	-	-	-	-	SRR31135525
Glc	2C	2	Chr	3,599,220	Δ1 bp	coding (800/993 nt)	*rpoS* →	SRR31135524
Glc	3A	2	Chr	284,048	C→T	R161C (CGT→TGT)	*HTH transcriptional regulator* →	SRR31135523
Glc	3B	2	Chr	3,598,766	(CTGGCGT)$_{1\rightarrow2}$	coding (346/993 nt)	*rpoS* →	SRR31135522
Glc	3C	2	-	-	-	-	-	SRR31135521
Glc	4A	2	Chr	3,598,679	(TCGCGCACTGCGTGGAG)$_{1\rightarrow2}$	coding (259/993 nt)	*rpoS* →	SRR31135520
Glc	4B	2	Chr	3,599,146	(A)$_{5\rightarrow4}$	coding (726/993 nt)	*rpoS* →	SRR31135519
Glc	4C	4	Chr	2,289,205	G→A	G506G (GGC→GGT)	*recQ* ←	SRR31135518
Glc	4C		Chr	3,598,713	Δ1 bp	coding (293/993 nt)	*rpoS* →	
Glc	5A	2	-	-	-	-	-	SRR31135517
Glc	5B	2	-	-	-	-	-	SRR31135515
Glc	5C	2	Chr	3,598,392	G→C	intergenic (+34/-29)	*nlpD* → / → *rpoS*	SRR31135514
Glc	6A	2	-	-	-	-	-	SRR31135513
Glc	6B	4	Chr	3,598,694	+CC	coding (274/993 nt)	*rpoS* →	SRR31135512
Glc	6C	2	-	-	-	-	-	SRR31135511
GEN	1A	2	Chr	1,714,698	T→G	*36Y (TAA→TAC)	*fimA* ←	SRR31135510
GEN	1A		Chr	2,242,150	T→G	I15L (ATC→CTC)	*trkH* ←	
GEN	1B	2	Chr	2,241,639	A→T	L185Q (CTG→CAG)	*trkH* ←	SRR31135509
GEN	1C	2	Chr	602,136	C→A	intergenic (-62/+1088)	*aroG* ← / ← *TetR family transcriptional regulator*	SRR31135508
GEN	1C		Chr	1,180,778	T→A	W329R (TGG→AGG)	*hyxB* →	
GEN	2A	2	Chr	2,331,982	Δ71 bp	coding (862-932/1104 nt)	*wecA* ←	SRR31135507
GEN	2A		Chr	3,331,595	T→G	S95R (AGT→AGG)	*sfaH* →	
GEN	2B	2	Chr	2,242,120	C→T	G25R (GGG→AGG)	*trkH* ←	SRR31135506
GEN	2C	2	Chr	1,714,700	A→T	*36K (TAA→AAA)	*fimA* ←	SRR31135504
GEN	3A	2	Chr	1,180,193	(GTT)$_{3\rightarrow2}$	coding (400-402/2298 nt)	*hyxB* →	SRR31135503
GEN	3A		Chr	2,332,830	(T)$_{7\rightarrow6}$	coding (84/1104 nt)	*wecA* ←	
GEN	3A		Plasmid	9,436	G→A	L236L (CTG→TTG)	*repA* ←	
GEN	3B	2	Chr	2,241,742	G→A	P151S (CCT→TCT)	*trkH* ←	SRR31135502
GEN	3C	2	Chr	1,180,193	(GTT)$_{3\rightarrow2}$	coding (400-402/2298 nt)	*hyxB* →	SRR31135501
GEN	3C		Plasmid	9,436	G→A	L236L (CTG→TTG)	*repA* ←	
GEN	4A	4	Chr	2,241,639	A→T	L185Q (CTG→CAG)	*trkH* ←	SRR31135500
GEN	4B	4	Chr	2,241,639	A→T	L185Q (CTG→CAG)	*trkH* ←	SRR31135499
GEN	4C	2	Chr	785,747	G→A	A219V (GCG→GTG)	*aroG* ←	SRR31135498
GEN	4C		Chr	3,240,668	(A)$_{8\rightarrow9}$	coding (3251/3342 nt)	*HAD family hydrolase* →	
GEN	5A	2	Chr	1,001,013	G→A	A20V (GCG→GTG)	*fetA* ←	SRR31135497
GEN	5A		Chr	2,242,120	C→T	G25R (GGG→AGG)	*trkH* ←	
GEN	5A		Plasmid	17,698	A→T	L931Q (CTG→CAG)	*traI* ←	
GEN	5B	2	Chr	1,180,778	T→A	W329R (TGG→AGG)	*hyxB* →	SRR31135496
GEN	5C	2	Chr	1,180,778	T→A	W329R (TGG→AGG)	*hyxB* →	SRR31135495
GEN	6A	2	Chr	1,180,193	(GTT)$_{3\rightarrow2}$	coding (400-402/2298 nt)	*hyxB* →	SRR31135493
GEN	6B	2	Chr	89,289	G→A	E266K (GAG→AAG)	*sapA* →	SRR31135492
GEN	6C	2	Chr	1,180,193	(GTT)$_{3\rightarrow2}$	coding (400-402/2298 nt)	*hyxB* →	SRR31135491
Glc+GEN	1A	2	-	-	-	-	-	SRR31135490
Glc+GEN	1B	2	Chr	3,599,171	C→T	Q251* (CAA→TAA)	*rpoS* →	SRR31135489
Glc+GEN	1C	4	-	-	-	-	-	SRR31135488
Glc+GEN	2A	2	Chr	3,598,623	+T	coding (203/993 nt)	*rpoS* →	SRR31135487
Glc+GEN	2A		Chr	3,942,188	T→G	Q74P (CAA→CCA)	*ipuB* ←	
Glc+GEN	2B	4	-	-	-	-	-	SRR31135486
Glc+GEN	2C	2	-	-	-	-	-	SRR31135485
Glc+GEN	3A	4	Chr	3,599,189	C→T	Q257* (CAG→TAG)	*rpoS* →	SRR31135484
Glc+GEN	3B	2	-	-	-	-	-	SRR31135482
Glc+GEN	3C	2	Chr	1,762,388	G→A	intergenic (+40/-76)	*yfcC* → / → *Arginine repressor*	SRR31135481
Glc+GEN	3C		Chr	3,599,189	C→T	Q257* (CAG→TAG)	*rpoS* →	
Glc+GEN	4A	2	Chr	4,494,257	C→A	R193S (CGC→AGC)	*dgcQ* →	SRR31135480
Glc+GEN	4B	2	Chr	1,762,420	A→T	intergenic (+72/-44)	*yfcC* → / → *Arginine repressor*	SRR31135479
Glc+GEN	4B		Chr	3,598,509	T→G	L30* (TTA→TGA)	*rpoS* →	
Glc+GEN	4C	2	-	-	-	-	-	SRR31135478
Glc+GEN	5A	2	-	-	-	-	-	SRR31135477
Glc+GEN	5B	4	Chr	3,598,688	Δ1 bp	coding (268/993 nt)	*rpoS* →	SRR31135476
Glc+GEN	5C	2	-	-	-	-	-	SRR31135475
Glc+GEN	6A	2	Chr	1,445,648	A→C	G667G (GGT→GGG)	*fhuA* ←	SRR31135474
Glc+GEN	6A		Chr	3,597,462	Δ6,506 bp		*[murein hydrolase activator NlpD] –[ygbL]*	
Glc+GEN	6B	2	-	-	-	-	-	SRR31135473
Glc+GEN	6C	2	-	-	-	-	-	SRR31135471
